# Third-hand smoke perception and awareness among medical students: a survey study

**DOI:** 10.1038/s41598-024-61636-9

**Published:** 2024-06-10

**Authors:** Aysun Aras, Mustafa Bayraktar

**Affiliations:** 1 Department of Public Health, Ataturk University Medical Faculty, Morphology Buildings, A-Block, 25100 Erzurum, Turkey; 2 Department of Family Medicine, Atatürk University, Medical Faculty, Erzurum, Turkey

**Keywords:** Awareness, Medical students, Preventive medicine, Public health, Third-hand smoke, Human behaviour, Public health

## Abstract

Third-hand smoke (THS) is tobacco smoke impurities that adhere to indoor materials such as clothing, dust, and surfaces and are released into the air. It is a major public health concern that, if unaddressed, could cause future harm. The aim of this study was to assess medical students’ knowledge of THS and to increase awareness. In March and April 2023, students enrolled in the medical programs at the School of Medicine for the 2022–2023 academic year were contacted using an online Google survey method and asked to complete the survey questions. The survey included questions on participants’ sociodemographic characteristics and the Beliefs about Third-Hand Smoke Scale (BATHS-T). The median age of the 351 students who voluntarily participated in the study was 20.0 (IQR = 2.0) years, 55.3% were female and 16% were smokers. The mean score of the answers given by the participants to the scale questions asking their level of knowledge about third-hand smoke was 35.3 ± 5.9. The least known question was “cigarette smoke particles can stay in a room for weeks”. The most frequently answered question was “breathing the air in a room where people smoked yesterday can damage the health of babies and children”. Scale scores were significantly higher for participants who did not have smokers living in their home, who did not allow smoking in their home, and who reported having information about passive smoking. Medical students had sufficient knowledge and awareness of third-hand smoke. Third-hand smoke should be included in training to increase knowledge and awareness of medical students as part of preventive medicine practice.

## Introduction

Tobacco use is the most important public health problem in the world, especially in developing countries^1^. Half of those who start and continue to smoke will die from smoking and, unfortunately, tobacco use is responsible for the deaths of more than 8 million people each year^2,3^. Physicians and other health professionals have an increasingly important role to play in reducing the risk of tobacco-related health problems at the societal level. Although the health effects of environmental tobacco smoke (ETS) are not yet fully understood, some studies have identified potential risks. This trend is of great concern, especially since children and people with respiratory diseases such as asthma are at greater risk from ETS exposure.

Indirect exposure to tobacco and tobacco products over a longer period of time is being considered. Many of the adverse effects include symptoms that are also caused by ETS, such as increased incidence of cough, asthma, upper and lower respiratory tract disease, lower respiratory tract infections, and increased severity of childhood pneumonia; ETS can also cause a variety of ear, nose and throat conditions, such as middle ear infections, sensorineural hearing loss and tonsillitis^4,5^.

Third-hand smoke (THS) is defined as a persistent residue of smoke that adheres to dust and surfaces in indoor environments and is re-emitted into the air, posing a risk to public health^6^. The compounds found in THS can damage DNA in human cells, and recognizing the presence of third-hand smoke is critical to minimizing this^7^. The most important difference, however, is that THS involves not only the inhalation route, but also the ingestion and topical routes. An individual may be exposed to THS pollutants through inhalation, ingestion and dermal absorption of pollutants found in smokers’ homes and, unfortunately, THS can affect people of all ages, from infants to the elderly, and is an important risk factor for the development of several cardiovascular and pulmonary diseases, including lung cancer^8^.

The level of knowledge of medical students, who are expected to have sufficient knowledge and awareness of the harms of smoking and THS, needs to be assessed and, if necessary, training and awareness-raising activities should be undertaken. In this context, this study aims to investigate medical students’ knowledge and perceptions of THS.

## Methods

### Study design and ethical approval

The study was designed as a cross-sectional, descriptive study. Prior to the study, ethical approval was obtained from the Atatürk University Faculty of Medicine Clinical Research Ethics Committee with the date and number 29.12.22-10/58. The study adhered to the ethical tenets of the Declaration of Helsinki.

### Setting and sample size

The study was conducted at a university medical school in March and April 2023. The study population consisted of the first 3-year medical students studying at Atatürk University Faculty of Medicine in the academic year 2022–2023. The total number of students in the faculty is 2312 and 1156 is considered as half of the number of students in the first 3 preclinical years and the sample size was aimed to cover all these students.

### Questionnaire

The study was prepared online, using the Google survey method, and the questionnaire form was delivered to the students and they were asked to complete it online. In the questionnaire form, they were first asked if they wanted to voluntarily participate in the study, and if they answered no, the survey ended. After answering yes, the questions appeared and could be answered. The first questions of the questionnaire asked about demographic characteristics, followed by the “Beliefs about Third-Hand Smoking (BATHS-T)” survey, the validity and reliability of which has been investigated in Turkey^9^. The nine-item survey has two sub-dimensions, health and permanence, with five-point Likert-type responses ranging from ‘strongly disagree’ to ‘strongly agree’. Participants score between 9 and 45 points.

### Statistical analysis

SPSS V23.0 (IBM, USA) program was used for statistical analysis of the data obtained from the study. The normal distribution of numerical data was evaluated by Kolmogorov–Smirnov test. Categorical data were presented as frequency and percentage, numerical data were presented as mean and standard deviation if normally distributed, median and interquartile range (IQR) if not normally distributed. For normally distributed numerical data, Student’s t-test was used to analyze two groups and ANOVA test was used to analyze more than three groups. For non-normally distributed numerical data, Mann–Whitney U test was used to analyze two groups and Kruskal–Wallis test was used to analyze more than three groups. In the whole study, *p* < 0.05 was taken for statistical significance.

### Ethics approval and consent to participate

The study was approved by the Ethics Committee of Erzurum Ataturk University School of Medicine (IRB 2022-10/58). Before participating, all individuals provided their informed consent. All research procedures adhered to the guidelines set by the Declaration of Helsinki.

## Results

A total of 374 participants (out of 1156 students, response rate 32,4%) completed the online survey as part of the study in the first 3 classes of medical students at the medical school. Of these, 9 people stated that they did not want to voluntarily participate in the study and answered “no” and were excluded from the study. Of the 365 people who volunteered, 14 people were excluded from the study due to duplicate responses, leaving a total of 351 people included in the study (inclusion rate 30.4%).

The median age of the participants was 20.0 (IQR = 2.0) years, 55.3% (n = 194) were female, 63.8% (n = 224) were medical students and 50.1% (n = 176) were first year students. Smoking was reported by 16% (n = 56), of whom 26 had been smoking for three years or more and 18 reported smoking immediately after waking. The demographic characteristics of the participants are shown in Table 1.

**Table 1:** Socio-demographic characteristics of the participants included in the study.

		Frequency	Percent
Sex	Male	157	44.7
	Female	194	55.3
Age (median, IQR)		20	2.0
With whom do you live?	At home with my family	215	61.3
	At home with a friend	19	5.4
	Home alone	20	5.7
	At home	97	27.6
Chronic disease	No	319	90.9
	Yes	32	9.1
Does anyone smoke at home?	No	181	51.6
	Yes	170	48.4
Do you smoke at home?	Never	154	43.9
	In some places	177	50.4
	Everywhere	20	5.7
	Total	351	100.0
Do you know about third-hand smoke?	No	300	85.5
	Yes	51	14.5
Do you smoke?	No	295	84.0
	Yes	56	16.0

**Figure 1:**
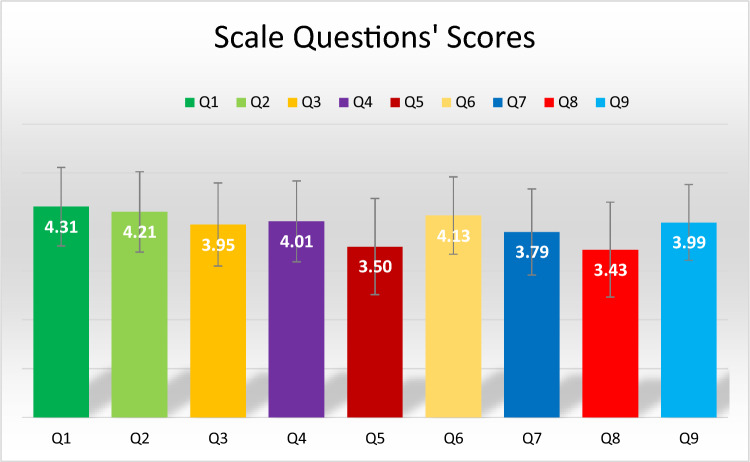
Mean scores of each question in the Questionnaire.

The mean score of the responses given by all participants to the scale questions asking about their level of knowledge of third-hand smoke was 35.3 ± 5.9 (median = 35.0, IQR = 7.0). The mean scores of participants’ responses to each question are shown in Fig. 1. According to this, the question with the lowest score, and therefore the least known question, was question 5, ‘Cigarette smoke particles can stay in a room for weeks’. The question with the highest score was question 1: “Breathing the air in a room where people smoked yesterday can damage the health of babies and children”. The responses and scale scores of the participants according to their socio-demographic characteristics are shown in Table 2. Although it was found that women scored higher according to gender, no statistical difference was found (*p* = 0.893). Similarly, no significant difference was found according to the age of the participants (*p* = 0.268).

**Table 2:** Scale scores and statistical comparisons of the participants according to their socio-demographic characteristics.

		N	Mean	Median	Std. deviation	Minimum	Maximum	*P*
Sex	Male	157	35.17	35.00	6.56	9	45	0.893*
	Woman	194	35.44	35.00	5.32	18	45	
Where do you live?	At home with my family	215	35.65	35.00	5.57	17	45	0.234^†^
	At home with a friend	19	33.05	33.00	6.39	22	45	
	Home alone	20	35.90	36.00	7.92	11	45	
	At dormitory	97	34.92	34.00	6.02	9	45	
Chronic disease	No chronic disease	319	35.40	35.00	5.98	9	45	0.213*
	Chronic disease	32	34.50	34.00	5.11	24	45	
Is there a smoker at home?	No smoker at home	181	35.83	36.00	5.78	16	45	0.042*
	There’s a smoker in the house	170	34.78	34.00	6.00	9	45	
Where to smoke in the house?	No smoking at home	154	36.14	36.00	5.84	16	45	0.014^†^
	In some places it is drunk	177	34.98	34.00	5.67	9	45	
	Drink everywhere	20	31.95	34.00	7.12	11	40	
Do you know anything about third-hand smoke?	I don’t know anything about third-hand smoke	300	35.10	35.00	5.88	9	45	0.026*
	I know about third-hand smoke	51	36.61	36.00	5.93	18	45	
Do you smoke?	I don’t smoke	295	35.49	35.00	5.51	16	45	0.478*
	I smoke cigarettes	56	34.39	35.00	7.62	9	45	
How long have you been smoking?	Never smoked	295	35.49	35.00	5.51	16	45	0.624^†^
	Less than one year	11	32.91	34.00	9.44	9	45	
	For one or two years	19	34.37	35.00	5.44	22	43	
	Three years and over	26	35.04	36.00	8.34	11	45	
Reason for smoking?	Stress	30	34.43	33.50	5.84	22	45	0.244^†^
	Once I started, I couldn’t stop	14	35.93	36.00	7.41	17	45	
	I learnt from my elders	5	39.00	37.00	5.79	32	45	
	Friends’ advice	4	29.50	35.00	13.82	9	39	
	For enjoying	3	25.67	32.00	12.74	11	34	
How much do you smoke?	I don’t smoke at all	295	35.49	35.00	5.51	16	45	0.904^†^
	Less than half a packet	25	34.24	34.00	7.77	9	45	
	Half to one packet	17	35.12	36.00	4.86	26	43	
	One to one and a half packs	14	33.79	34.50	10.21	11	45	
Do you smoke as soon as you wake up in the morning?	No. I don’t smoke as soon as I wake up in the morning	38	35.61	35.00	5.70	22	45	0.346*
	Yeah. I’ll drink it as soon as I wake up in the morning	18	31.83	34.50	10.34	9	45	

*Mann–Whitney U test.

^†^Kruskal–Wallis test.

As expected, the scale score was higher for participants who reported having information about passive smoking, and a statistically significant difference was found (*p* = 0.026). Although the score of non-smokers was higher, it was not statistically different from that of smokers (*p* = 0.478). Participants who reported that there was no smoker in their home had a higher scale score and this was statistically significant (*p* = 0.042). Similarly, participants who reported that smoking was not allowed in the home had a higher scale score and this was found to be significant (*p* = 0.014).

One of the questions asked in the questionnaire to determine the dependence of smokers was whether they smoked when they got up in the morning, and the scale score of those who did not smoke when they got up in the morning was higher, but not statistically different (*p* = 0.346).

## Discussion

The World Health Organization (WHO) estimates that 21% of adults worldwide (36% of men and 7% of women) are current smokers^1^. Beyond the active smoking individual, the surrounding community is also vulnerable to the harmful effects of secondhand and thirdhand smoke. Being aware of all these negative effects of smoking will not only reduce smoking rate, but also increase self-protection efforts, which will increase society’s health.

In our study, medical students’ knowledge of THS was assessed and scored 35.3 out of 45. This was considered to be an adequate result. Male students were more likely than female students to be smokers and more aware of THS. However, when asked about THS, 85% of the participants said they had no knowledge of THS. Being aware of THS but claiming not to know about THS may be due to not knowing or hearing the nomenclature. Similarly, a study found that six out of ten young adults living in California had never heard of THS^10^. But, a meta-analysis of 12 studies found that society’s knowledge of the harmful effects of THS was 80.1%^11^. This was also a clue to the higher consciousness, but not knowing its existence.

A review highlighted the need to shed light on the issue and include it in public health discussions, noting that little is known about THS and few people are aware of its existence and potential health effects^12^. Of course, with more knowledge comes more awareness and more efforts at prevention, and a healthier society. A study investigating levels of THS knowledge concluded that community THS education interventions are likely to increase the prevalence of smoke-free environments, thereby minimizing the potential adverse health effects associated with THS exposure. It has been suggested that THS may affect indoor air quality and that long-term exposure may have adverse effects on respiratory problems, cancer risk and general health^13^. In a study that looked at these health issues and examined adults’ knowledge and perception of the risk of THS, it was found that awareness was very low^14^.

In our study, the scale score of participants who reported having information about THS was high and a statistically significant difference was found. The level of knowledge of THS among students on a university campus was found to be slightly low at 63.3%^15^. However, a study of pregnant women found high levels of THS knowledge, similar to our study, and concluded that exposure to THS decreased as education increased^16^.

The level of knowledge of health professionals needs to be higher than that of society, and in a study by Darlow et al.^17^ health professionals believed that smoking affected the quality of parenting and reported that they did not smoke at home. However, in a multinational study investigating health professionals’ knowledge of THS, almost two out of three participating health professionals did not know what THS was, and the authors concluded that education should be provided^18^. This shows that there is a need to raise awareness and knowledge levels while they are still at school, which, as our study shows, highlights the need for increased efforts in this direction in the education system.

## Conclusion

THS is an important area of research to understand the potential health effects of cigarette smoke residues. With further research, the risks of THS can be better understood and preventive measures can be taken accordingly. Therefore, ongoing research and awareness efforts are important to understand the health effects of THS and to improve public health.

In this study, investigating the awareness of THS among medical students, who represent the young population and who will play an important role in the fight against smoking in the future, may lead to the development of awareness. There is a need to investigate the level of knowledge of medical students, who should have a high level of knowledge and awareness of the harms of smoking and who will play an important role in raising public awareness of this issue.

### Limitations

This study has several limitations. First, the study population was the preclinical, first three years medical students, not covers all medical faculty students. Therefore, it may not reflect the knowledge level of all medical students. Second, the response rate of the medical students in our study was considerably low, and so the sample size can be considered small. Also, the study was conducted in a single center, all of which may limit the generalizability of the results. Third, the study was conducted using an online survey method with a self-report nature, therefore the responses may not reflect the true level of knowledge. However, since the study period was during the COVID-19 pandemic, a time when students were already accustomed to online learning, it may have influenced their receptivity to the online medium. Future research is needed that looks at the impact of cigarettes on everyone.

## Data Availability

The datasets used and/or analyzed during the current study available from the corresponding author on reasonable request.
